# Age-stratified comparison of clinical outcomes between medical and surgical treatments in patients with unilateral primary aldosteronism

**DOI:** 10.1038/s41598-021-86290-3

**Published:** 2021-03-25

**Authors:** Ryo Nakamaru, Koichi Yamamoto, Hiroshi Akasaka, Hiromi Rakugi, Isao Kurihara, Takashi Yoneda, Takamasa Ichijo, Takuyuki Katabami, Mika Tsuiki, Norio Wada, Tetsuya Yamada, Hiroki Kobayashi, Kouichi Tamura, Yoshihiro Ogawa, Junji Kawashima, Nobuya Inagaki, Megumi Fujita, Minemori Watanabe, Kohei Kamemura, Shintaro Okamura, Akiyo Tanabe, Mitsuhide Naruse, JPAS/JRAS Study Group, Hiroshi Itoh, Hisashi Fukuda, Hironobu Umakoshi, Yui Shibayama, Masanori Murakami, Takanobu Yoshimoto, Tatsuya Haze, Masakatsu Sone, Katsutoshi Takahashi, Yuichi Matsuda, Hirotaka Shibata, Michio Otsuki, Yuichi Fujii, Atsushi Ogo, Shozo Miyauchi, Toshihiko Yanase, Tomoko Suzuki, Takashi Kawamura, Mai Asano, Tomikazu Fukuoka, Tatsuya Kai, Shoichiro Izawa, Yuichiro Yoshikawa, Shigeatsu Hashimoto, Masanobu Yamada, Ryuichi Sakamoto, Yoshiro Chiba, Ryuji Okamoto, Kenji Oki, Daisuke Yabe

**Affiliations:** 1 Department of Geriatric and General Medicine, Osaka University Graduate School of Medicine, 2-2, Yamadaoka, Suita, 5650871 Japan; 2 Department of Endocrinology, Metabolism and Nephrology, School of Medicine, Keio University, Tokyo, Japan; 3 Department of Health Promotion and Medicine of the Future, Graduate School of Medical Science, Kanazawa University, Kanazawa, Japan; 4 Department of Diabetes and Endocrinology, Saiseikai Yokohamashi Tobu Hospital, Yokohama, Japan; 5 Division of Metabolism and Endocrinology, Department of Internal Medicine, St. Marianna University School of Medicine Yokohama City Seibu Hospital, Yokohama, Japan; 6 Department of Endocrinology and Metabolism, National Hospital Organization Kyoto Medical Center, Kyoto, Japan; 7 Department of Diabetes and Endocrinology, Sapporo City General Hospital, Sapporo, Japan; 8 Department of Molecular Endocrinology and Metabolism, Graduate School of Medical and Dental Sciences, Tokyo Medical and Dental University, Tokyo, Japan; 9 Division of Nephrology, Hypertension and Endocrinology, Nihon University School of Medicine, Tokyo, Japan; 10 Department of Medical Science and Cardiorenal Medicine, Yokohama City University Graduate School of Medicine, Yokohama, Japan; 11 Department of Medicine and Bioregulatory Science, Graduate School of Medical Sciences, Kyushu University, Fukuoka, Japan; 12 Department of Metabolic Medicine, Faculty of Life Sciences, Kumamoto University, Kumamoto, Japan; 13 Department of Diabetes, Endocrinology and Nutrition, Kyoto University Graduate School of Medicine, Kyoto, Japan; 14 Division of Nephrology and Endocrinology, University of Tokyo, Tokyo, Japan; 15 Department of Endocrinology and Diabetes, Okazaki City Hospital, Okazaki, Japan; 16 Department of Cardiology, Shinko Hospital, Kobe, Japan; 17 Department of Endocrinology, Tenri Hospital, Tenri, Japan; 18 Department of Diabetes, Endocrinology and Metabolism, National Center for Global Health and Medicine, Tokyo, Japan; 19 Endocrine Center, Ijinkai Takeda General Hospital, Kyoto, Japan; 20 Division of Metabolism, Showa General Hospital, Tokyo, Japan; 21 Department of Cardiology, Sanda City Hospital, Sanda, Japan; 22 Department of Endocrinology, Metabolism, Rheumatology and Nephrology, Faculty of Medicine, Oita University, Yufu, Japan; 23 Department of Metabolic Medicine, Osaka University Graduate School of Medicine, Suita, Japan; 24 Department of Cardiology, JR Hiroshima Hospital, Hiroshima, Japan; 25 Clinical Research Institute, National Hospital Organization Kyusyu Medical Center, Fukuoka, Japan; 26 Department of Internal Medicine, Uwajima City Hospital, Uwajima, Japan; 27 Department of Endocrinology and Diabetes Mellitus, Faculty of Medicine, Fukuoka University, Fukuoka, Japan; 28 Department of Public Health, School of Medicine, International University of Health and Welfare, Narita, Japan; 29 Department of Preventive Services, Kyoto University School of Public Health, Kyoto, Japan; 30 Department of Endocrinology and Metabolism, Kyoto Prefectural University of Medicine, Kyoto, Japan; 31 Department of Internal Medicine, Matsuyama Red Cross Hospital, Matsuyama, Japan; 32 Department of Cardiology, Saiseikai Tondabayashi Hospital, Tondabayashi, Japan; 33 Department of Endocrinology and Metabolism, Tottori University Hospital, Yonago, Japan; 34 Department of Endocrinology and Diabetes Mellitus, Misato Kenwa Hospital, Misato, Japan; 35 Department of Diabetes, Endocrinology and Metabolism, Fukushima Medical University Hospital, Fukushima, Japan; 36 Department of Medicine and Molecular Science, Gunma University Graduate School of Medicine, Maebashi, Japan; 37 Department of Diabetes and Endocrinology, Saiseikai Fukuoka General Hospital, Fukuoka, Japan; 38 Endovascular Treatment Group, Mito Saiseikai General Hospital, Mito, Japan; 39 Department of Cardiology and Nephrology, Mie University Graduate School of Medicine, Tsu, Japan; 40 Department of Molecular and Internal Medicine, Graduate School of Biomedical and Health Sciences, Hiroshima University, Hiroshima, Japan; 41 Department of Diabetes and Endocrinology, Gifu University Graduate School of Medicine, Gifu, Japan

**Keywords:** Endocrinology, Endocrine system and metabolic diseases

## Abstract

Although adrenalectomy (ADX) is an established treatment for unilateral primary aldosteronism (uPA), the influence of age on the surgical outcomes is poorly understood. Therefore, we aimed to elucidate how age affects the clinical outcomes after treatments. We analyzed 153 older (≥ 65 years) and 702 younger patients (< 65 years) with uPA, treated either with ADX or mineralocorticoid receptor antagonist (MRA) in the Japan PA Study, and compared the estimated glomerular filtration rate (eGFR) or blood pressure over a 36-month period after treatments. ADX-treated patients showed severer biochemical indicators than MRA-treated patients. During 6 and 36 months, the eGFR decreased more prominently in older but not in younger patients with ADX than in those with MRA, which remained significant after adjustment with the inverse probability of treatment weighting (IPTW). There was a significant interaction between the age-groups and the treatment choices in the change of the eGFR with IPTW-adjusted analysis. The post-treatment dose of antihypertensive medication was lower in younger and higher in older patients with ADX than those with MRA. The clinical benefit of ADX differed between younger and older patients with uPA. These findings indicate the need for further validation on whether ADX can benefit older patients with uPA.

## Introduction

Primary aldosteronism (PA), a major cause of secondary hypertension^1–3^, increases the risk of cardiovascular disease (CVD) as well as renal disease, via activation of the mineralocorticoid receptor (MR)^4–10^. Indeed, inappropriate aldosterone secretion is known to play a role in renal injury development^11^. The current guidelines recommend adrenalectomy (ADX) for unilateral PA (uPA), or MR antagonists (MRAs) for bilateral PA, as PA-specific treatments^1^. However, the effect of ADX on clinical outcome varies primarily depending on the baseline characteristics of patients including sex, obesity, and age^12–14^.

The Japan PA Study (JPAS) investigation group reported that remission of hypertension or reduction of antihypertensives shortly after ADX (6 or 12 months) was limited in older patients (≥ 65 years old) compared with that in younger patients^14^. We found that the appearance of renal impairment (chronic kidney disease (CKD) ≥ stage 3b) shortly after ADX was more frequent in older patients. Nevertheless, it remains unknown whether poorer clinical outcomes after ADX in older patients are attributed to different treatment benefits or are a simple reflection of different background characteristics between older and younger patients. To answer this question, it is necessary to compare the patients with ADX to those with the different treatment choice, namely medical treatment with MRA. In this study, we analyzed patients with uPA, treated with either ADX or MRA, to clarify whether the benefit of ADX to renal function and blood pressure (BP) compared to MRA differs between older and younger patients with uPA.

## Methods

### Study population and follow-up after specific treatments

**Figure 1:**
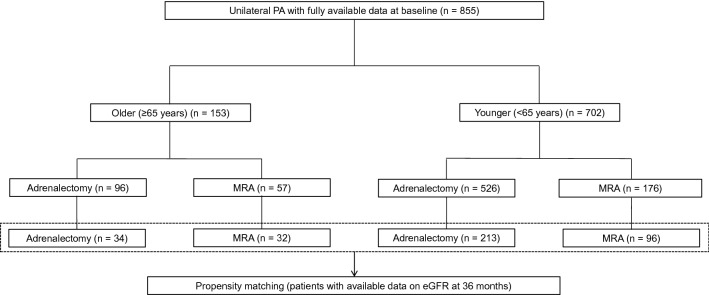
Study flowchart. BP, blood pressure; eGFR, estimated glomerular filtration rate; MRA, mineralocorticoid receptor antagonist; PA, primary aldosteronism.

This was a retrospective observational study that was part of the JPAS. The data of patients aged 20–90 years old with PA who underwent adrenal venous sampling (AVS) at 41 referral centers in Japan between January 2006 and December 2018 were collected as described previously^14–19^. Diagnosis of PA was based on the Japanese guidelines^20,21^. Of 1039 patients with uPA and the fully available data at baseline, 184 patients without follow up data on BP or eGFR were excluded. We then analyzed 153 older (≥ 65 years) and 702 younger patients (< 65 years) (Fig. 1). Both collected BP and estimated glomerular filtration rate (eGFR) were analyzed at baseline, 6, 12, or 36 months after performing an ADX or initiating MRA treatments. Additionally, we extracted 66 older and 309 younger patients with available eGFRs at 36 months for propensity score-matched analysis using the inverse probability of treatment weighting (IPTW) (Fig. 1). The decision on whether to perform an ADX or initiate MRA treatment was dependent on the judgement of patients and their attending physicians following classification of the PA subtype. Further, the dose or class of antihypertensives were decided by the attending physician at each center^17^.

### Analysis of patient characteristics and clinical outcomes

Each patient’s data were obtained from the medical records of each referral center. The BP levels in the seated position at outpatient clinics were obtained from medical records. The serum creatinine was measured by the enzyme method. eGFR was calculated using the following equation established for the Japanese population: eGFR (ml/min/1.73 m^2^) = 194 × serum creatinine^−1.094^ × age^−0.287^ (× 0.739 for female patients)^22^. Plasma aldosterone concentration (PAC) and plasma renin activity were measured in the supine position, using commercially available kits, as detailed in previous JPAS reports^14–18^. Hypokalemia or hyperkalemia was defined as a serum potassium concentration < 3.5 mEq/L or use of a potassium supplement, or as a serum potassium concentration > 5.0 mEq/L, respectively. The presence of proteinuria was defined as a positive reaction in the urine dipstick test. Clinical or biochemical success after ADX was assessed using the Primary Aldosterone Surgical Outcome criteria^13^. Briefly, complete biochemical success was defined as a normalization of the aldosterone-to-renin ratio (< 200) in the absence of hypokalemia. In addition, complete clinical success was defined as a normal BP without antihypertensive medication, whereas partial clinical success was defined as the same BP as before adrenalectomy with less antihypertensive medication or a reduction in BP with either the same or less antihypertensive medication. Antihypertensive medication was expressed as defined daily dose (DDD)^13^. In this study, composite cardiovascular events were defined as myocardial infarction, angina, stoke, heart failure, cardiac arrhythmias, peripheral artery diseases, or unplanned hospitalization related to any other cardiovascular diseases.

### Analysis of AVS

The lateralization index (LI) was calculated by dividing the aldosterone to cortisol ratio on the dominant side with that on the nondominant side by AVS with cosyntropin stimulation. The contralateral ratio was calculated by dividing the aldosterone to cortisol ratio in the non-dominant side to that in the inferior vena cava. The details of the AVS procedure were previously described^23^. The LI > 4 or 2 < LI ≤ 4 and contralateral ratio < 1 was defined as uPA^24,25^.

### Statistical analysis

Continuous variables were expressed as mean ± standard deviation or as the median (interquartile range). Categorical variables were expressed as absolute frequencies and percentages for categorical variables^16–19^. Differences in parametric and non-parametric variables were assessed by the Student’s t-test and Mann–Whitney U test, respectively^16–19^. The significance of differences between independent categorical variables was assessed using the Chi-squared test or Fisher’s exact test^16–19^. We calculated the Pearson correlation coefficients between the percentage change in eGFR from baseline to 36-month after PA-specific treatments in ADX or MRA group. We used mixed effects models for repeated measures to analyze the differences in the effect of PA-specific treatments on the temporal change in eGFR and systolic BP. We compared the differences in temporal changes of eGFRs between treatment choices (ADX or MRA) during a 3-year period, with or without the use of IPTW. To calculate the propensity scores, we used a logistic regression using gender, age, body mass index, systolic BP, log-transformed PAC, eGFR, the DDD of antihypertensives, and the presence of hypokalemia at baseline as covariates, as well as the treatment choices as a dependent variable. Weighting, with 1/propensity score in ADX-treated patients and 1/(1-propensity score) in MRA-treated patients, was performed to estimate the average treatment effect. The standardized difference was calculated to evaluate the balance of each confounding factor between the treatments, and the standardized difference of < 0.1 was considered well-balanced. A generalized linear model was used to evaluate the percent change in eGFRs in younger or older ADX-treated patients compared to MRA-treated patients with either unadjusted or IPTW-adjusted data. Finally, the p-values were calculated to assess the interaction between the age-groups and the treatment choices in the percent change in the eGFRs. p < 0.05 were considered significant. All statistical analyses were performed using JMP 15.3.0 software (SAS Institute, Cary, NC, USA) and SPSS statistics 27 (IBM Corporation, Armonk, NY, USA).

### Ethics

The study was conducted according to the guidelines for clinical studies published by the Ministry of Health and Labor, Japan, and it was approved by the ethics committee of the National Hospital Organization Kyoto Medical Center, as the project leader center, and by the institutional ethics committees of the participating centers. Informed consent was obtained in the form of opt-out on the web-site of each referral center. This observational study was registered at UMIN ID 000018756.

## Results

### Correlations between age at baseline and decline in eGFR after the treatments of uPA

**Figure 2:**
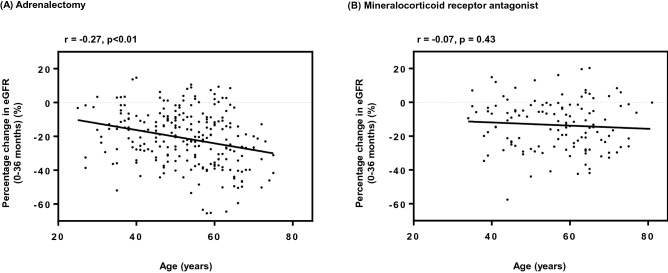
Correlations between age at baseline and decline in estimated glomerular filtration rate during 36 months following primary aldosteronism-specific treatment. (**A**) Adrenalectomy, (**B**) Mineralocorticoid receptor antagonist. The significant correlation was observed in 247 patients with ADX (r =  − 0.27, p < 0.01) but not in 128 patients with MRA (r =  − 0.07, p = 0.43). ADX, adrenalectomy; eGFR, estimated glomerular filtration rate; MRA, mineralocorticoid receptor antagonist.

We first analyzed the correlation between age at baseline and decline in eGFR during 36 months after medical or surgical treatment of uPA. As shown in Fig. 2, the significant correlation was observed in 247 patients with ADX (r =  − 0.27, p < 0.01) but not in 128 patients with MRA (r =  − 0.07, p = 0.43). Thereafter, we performed age-stratified analysis by dividing the patients into the younger (< 65 years) and the older (≥ 65 years) groups.

### Comparison of patient characteristics between patients with ADX and MRA in older and younger patients

The patient characteristics are shown in Table 1. In both age groups, patients with ADX had more severe biochemical features of uPA than those treated with MRA, including a higher level of LI and PAC, and prevalence of hypokalemia at the baseline. The eGFR level at the baseline was significantly higher in patients with ADX than in those treated with MRA in the younger group, whereas there was no significant difference in the older group.

**Table 1:** Comparison of patient characteristics between patients with ADX and MRA.

	Older (Age ≥ 65 y)			Younger (Age < 65 y)		
	ADX N = 96	MRA N = 57	p-value	ADX N = 526	MRA N = 176	p-value
**Baseline characteristics**						
Age, years	67 (65–70)	68 (66–72)	0.18	50 (40–57)	53 (46–60)	< 0.01
Female, n (%)	37 (39)	31 (54)	0.07	263 (50)	67 (38)	< 0.01
BMI, kg/m^2^	23.7 ± 3.69	23.6 ± 3.27	0.99	24.2 ± 4.11	25.4 ± 4.16	< 0.01
SBP, mmHg	143.3 ± 18.3	142.1 ± 15.8	0.69	141.3 ± 18.9	142.6 ± 17.5	0.42
DBP, mmHg	81.1 ± 12.5	81.0 ± 11.9	0.95	87.7 ± 12.1	88.3 ± 12.7	0.62
eGFR, mL/min/1.73m^2^	67.4 ± 17.4	66.6 ± 17.4	0.80	81.6 ± 22.9	76.4 ± 17.3	< 0.01
Duration of HT, years	19 (10–26)	15 (6–23)	0.052	7 (2–12)	7 (3–15)	0.51
Hypokalemia, n (%)	77 (80)	28 (49)	< 0.01	409 (78)	91 (52)	< 0.01
Proteinuria, n (%)	22 (24) (n = 91)	17 (31) (n = 55)	0.37	90 (18) (n = 497)	25 (15) (n = 167)	0.41
**PA characteristics**						
Lateralization index	12.7 (7.5–27.3)	6.9 (4.2–12.8)	< 0.01	12.1 (6.1–26.8)	6.2 (3.4–13.4)	< 0.01
PRA, ng/mL/h						
Baseline	0.2 (0.1–0.4)	0.2 (0.1–0.4)	0.53	0.3 (0.2–0.4)	0.3 (0.2–0.5)	0.01
6 or 12 months	0.7 (0.4–1.4) (n = 70)	0.4 (0.3–1.3) (n = 25)	< 0.01	1.1 (0.5–2.2) (n = 409)	0.8 (0.4–1.6) (n = 122)	< 0.01
PAC, pg/mL						
Baseline	281 (181–396)	186 (139–286)	< 0.01	316 (211–469)	195 (147–314)	< 0.01
6 or 12 months	80 (57–123) (n = 74)	299 (170–465) (n = 28)	< 0.01	99 (72–136) (n = 421)	268 (183–391) (n = 128)	< 0.01

Values are mean ± standard deviation, median (interquartile range), or n (%). ADX, adrenalectomy; ARR, aldosterone-renin ratio; BMI, body mass index; DBP, diastolic blood pressure; DDD, defined daily dose; eGFR, estimated glomerular filtration rate; HT, hypertension; MRA, mineralocorticoid receptor antagonist; PAC, plasma aldosterone concentration; PRA, plasma renin activity; SBP, systolic blood pressure.

### Clinical outcomes after the treatments with ADX or MRA in older and younger patients

The prevalence of complete clinical success after ADX that indicates the cure of hypertension^13^ was significantly higher in younger patients than older patients (Table 2). In contrast, the prevalence of complete biochemical success after ADX that indicates the cure of PA^13^ was similarly high both in younger and older patients (Table 2). There was no significant difference in the post-treatment occurrence of the composite cardiovascular events for 36 months between patients with MRA and with ADX in both age-groups (Table 2).

**Table 2:** Comparison of clinical outcomes.

	Older (Age ≥ 65 y)			Younger (Age < 65 y)		
	ADX N = 96	MRA N = 57	p-value	ADX N = 526	MRA N = 176	p-value
Complete clinical success, n (%)	16 (37) (n = 43)	–	–	185 (55) (n = 335)	–	0.02
Partial clinical success, n (%)	13 (30) (n = 43)	–	–	93 (28) (n = 335)	–	
Absent clinical success, n (%)	14 (33) (n = 43)	–	–	57 (17) (n = 335)	–	
Complete biochemical success, n (%)	53 (77) (n = 69)	–	–	316 (77) (n = 410)	–	0.96
Hyperkalemia, n (%)	15 (16) (n = 94)	1 (1.8) (n = 56)	< 0.01	28 (5.5) (n = 514)	3 (1.7) (n = 175)	0.05
Proteinuria, n (%) (6 or 12 months)	7 (11) (n = 62)	6 (17) (n = 35)	0.54	29 (8.8) (n = 331)	15 (13) (n = 117)	0.20
Cardiovascular event, n (%) (during 36 months)	6 (6.7) (n = 90)	6 (11) (n = 54)	0.37	6 (1.2) (n = 499)	5 (3.0) (n = 169)	0.16

Values are n (%). The p-values of clinical success or complete biochemical success represent the comparison between older and younger patients treated with ADX. ADX, adrenalectomy; MRA, mineralocorticoid receptor antagonist.

### Temporal changes in renal function after PA-specific treatment

**Figure 3:**
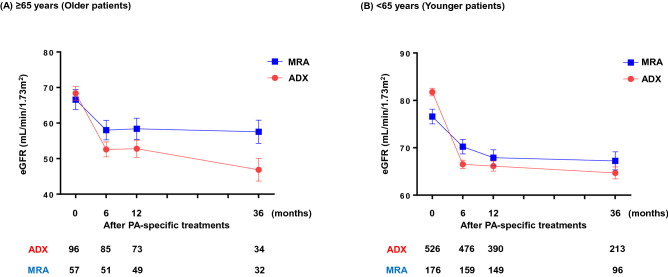
Mean estimated glomerular filtration rate from baseline to 36-months following primary aldosteronism-specific treatment. (**A**) ≥ 65 years (Older patients), (**B**) < 65 years (Younger patients). A mixed effects model for repeated measures revealed that eGFR reduced more prominently with ADX than with MRA in both age groups during 3 years (p < 0.001) (**A**,**B**). However, the decline in eGFR from 6 to 36 months was more prominent in patients with ADX than in those with MRA in older (p = 0.039) (**A**), but not in younger patients (p = 0.26) (**B**). Error bars indicate the standard error. ADX, adrenalectomy; eGFR, estimated glomerular filtration rate; MRA, mineralocorticoid receptor antagonist; PA, primary aldosteronism.

The temporal changes in eGFR following PA-specific treatment are shown in Fig. 3. Analysis using a mixed effects model revealed that ADX reduced eGFR more prominently than that with MRA in both age groups during 36 months (p < 0.001). In contrast, the eGFR decreased to a greater extent in older patients with ADX than MRA-treated patients between the 6 to 36 months after treatment (p = 0.039); however, this difference was not observed in younger patients during this time period (p = 0.26) (Fig. 3). There was no significant difference in the post-treatment prevalence of proteinuria between ADX and MRA in both younger and older patients who had data at 6 or 12 months (Table 2).

### Comparison of BP reduction after PA-specific treatments

**Figure 4:**
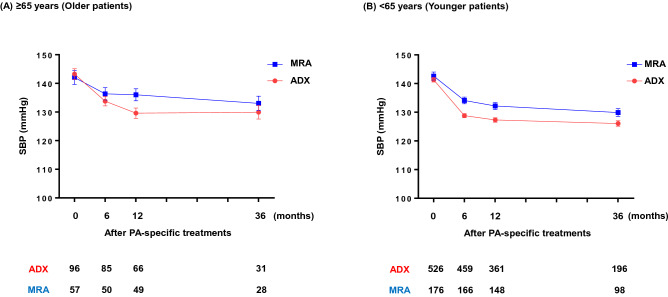
Mean systolic blood pressure from baseline to 36-months following primary aldosteronism-specific treatment. (**A**) ≥ 65 years (Older patients), (**B**) < 65 years (Younger patients). A temporal change in systolic BP during 36 months was not significantly different between ADX and MRA in both older (p = 0.19) (**A**) and younger patients (p = 0.08) (**B**) using a mixed effects model for repeated measures. Error bars indicate the standard error. ADX, adrenalectomy; MRA, mineralocorticoid receptor antagonist; PA, primary aldosteronism; SBP, systolic blood pressure.

The temporal change in BP after treatment was not significantly different between ADX and MRA in both older (p = 0.19) and younger (p = 0.08) patients using the mixed effects model (Fig. 4). DDD in antihypertensives except for MRAs was lower after the treatment with ADX than with MRA in younger patients (Table 3). In contrast, older patients with ADX received higher DDD of antihypertensives except for MRAs than those with MRA after treatment (Table 3).

**Table 3:** Comparison of antihypertensive therapies between patients with ADX and MRA.

	Older (Age ≥ 65 y)			Younger (Age < 65 y)		
	ADX N = 96	MRA N = 57	p-value	ADX N = 526	MRA N = 176	p-value
**Baseline**						
Number	2 (1–2)	1 (1–2)	< 0.01	1 (1–2)	1 (1–2)	0.28
DDD	1.5 (1.3–2.4)	1.3 (1.0–2.0)	0.14	1.3 (1.0–2.0)	1.3 (1.0–2.0)	0.31
**6 or 12 months (after ADX or MRA)**						
Number	1 (1–2)	2 (2–3)	< 0.01	1 (0–1)	2 (2–2)	< 0.01
DDD	1.0 (1.0–1.5) (n = 68)	2.0 (1.0–2.5)	< 0.01	0 (0–1.0) (n = 432)	2.0 (1.0–3.0)	< 0.01
DDD (MRA)	–	1.0 (0.7–2.0)	–	–	1.0 (0.7–2.0)	0.88
DDD (Except for MRA)	1.0 (0–1.5) (n = 68)	0.3 (0–1.0)	0.03	0 (0–1.0) (n = 432)	0.5 (0–1.3)	0.01
MRA						
Spironolactone, n (%)	–	16 (28)	–	–	59 (34)	0.44
Eplerenone, n (%)	–	41 (72)	–	–	117 (66)	
ARB/ACE-I, n (%)	18 (19)	5 (8.8)	0.11	68 (13)	28 (16)	0.32
CCB, n (%)	75 (78)	46 (81)	0.70	281 (53)	132 (75)	< 0.01
Diuretics, n (%)	1 (1.0)	1 (1.8)	0.71	2 (2.3)	4 (2.3)	0.99
Alfa-blocker, n (%)	10 (10)	7 (12)	0.72	39 (7.4)	16 (9.1)	0.52
Beta-blocker, n (%)	8 (8.3)	3 (5.3)	0.48	23 (4.4)	10 (5.7)	0.48

Values are median (interquartile range) or n (%). The p-values of DDD and kinds of MRA represent the comparison between older and younger patients treated with MRA. ACE-I, angiotensin converting enzyme inhibitor; ADX, adrenalectomy; ARB, angiotensin II receptor blocker; CCB, calcium channel blocker; DDD, defined daily dose.

### Propensity scores-adjusted comparison of the eGFR over 36-month period between treatments

Finally, to reduce the selection bias for a PA-specific treatment, we adjusted with IPTW using the PS to analyze the available eGFR at 36 months among patients (Fig. 1). Gender, age, body mass index, systolic BP, log-transformed PAC, eGFR, the DDD of antihypertensives, and the presence of hypokalemia at baseline were used as covariates to calculate the PS. All of the standardized differences of IPTW-adjusted covariates between the treatments were less than 0.1, indicating that the IPTW sufficiently balanced the patient’s backgrounds between treatments (Table 4). A generalized linear model with the IPTW-adjusted analysis indicated that the percent change in the eGFR from the baseline to 36-month in ADX was 17.0% or 4.7% greater than the MRA treatment in older or younger patients, respectively (Table 4). There was a significant interaction between the age-groups and the PA treatments in the percent change of the eGFR, indicating that the influence of the treatment of choice on the percent change of the eGFR differed between younger and older patients. When the 36-month period used to calculate the eGFR was divided into the initial (0–6 months) and late (6–36 months) phases, we found that ADX enhanced the initial phase change in eGFR compared to MRA in both age groups; however, the interaction between the age groups and the treatment choices was not significant. In contrast, the late phase eGFR in ADX was 9.4% greater than that in MRA in older patients with IPTW-adjusted analysis; however, there was no treatment-associated difference in the younger patients. There was a significant interaction between the age-groups and the treatment choices in the late phase change in eGFR.

**Table 4:** Differences in the percentage change of eGFR with ADX compared to that with MRA in generalized linear models with or without the use of IPTW.

	Older			Younger			p-value for interaction
	Mean	95% CI	p-value	Mean	95% CI	p-value	
**From baseline to 36 months, %**							
Non-adjusted	− 19.4	− 26.2 to − 12.6	< 0.001	− 5.95	− 9.55 to − 2.34	0.001	< 0.001
IPTW-adjusted	− 17.0	− 24.3 to − 9.6	< 0.001	− 4.70	− 8.48 to − 0.92	0.015	0.003
**From baseline to 6 months, %**							
Non-adjusted	− 11.4	− 19.8 to − 3.0	0.008	− 8.01	− 11.7 to − 4.36	< 0.001	0.440
IPTW-adjusted	− 7.20	− 15.9 to 1.5	0.107	− 6.12	− 10.4 to − 1.83	0.005	0.132
**From 6 to 36 months, %**							
Non-adjusted	− 7.90	− 14.4 to − 1.40	0.017	2.87	− 0.95 to 6.70	0.141	0.013
IPTW-adjusted	− 9.44	− 16.1 to − 2.75	0.006	2.10	− 2.67 to 6.87	0.389	0.011

Gender, age, body mass index, SBP, log-transformed PAC, eGFR, DDD of antihypertensives, and the presence of hypokalemia at baseline were used as covariates. Standardized differences of each covariate between the treatments before (unadjusted) and after the adjustment with IPTW (IPTW-adjusted) are as follows: Gender, 0.10 and 0.01; age, 0.35 and 0.02; body mass index, 0.17 and 0.05; SBP, 0.24 and 0.01; log-transformed PAC, 0.54 and 0.03; eGFR, 0.15 and 0.05; the DDD of antihypertensives, 0.06 and 0.04; the presence of hypokalemia, 0.50 and 0.00, respectively. p-value for the interaction between the age groups and the treatment choices (ADX or MRA). ADX, adrenalectomy; CI, confidence interval; DDD, daily defined dose, eGFR, estimated glomerular filtration rate; IPTW, inverse probability of treatment weighting; MRA, mineralocorticoid receptor antagonist; PAC, plasma aldosterone concentration; SBP, systolic blood pressure.

## Discussion

To the best of our knowledge, this is the first study to investigate the impact of age on the clinical outcome of ADX in comparison to MRA in patients with uPA. The primary findings of the present study are as follows: the significant correlation between the change in eGFR (0–36 months) and age was observed in patients with ADX, but not in patients with MRA; the ADX treatment lowered the eGFR compared with the MRA treatment during a 36-month period in both older and younger patients; a late phase (6–36 months) decline in the eGFR was higher with the ADX treatment than with the MRA treatment in older patients but not in younger patients; the post-treatment dose of antihypertensive medication was lower in younger and higher in older patients with ADX than those with MRA; the late phase decline in eGFR with the ADX treatment was greater than that with the MRA treatment in older patients but not in younger patients with the IPTW-adjusted analysis; there was a significant interaction between the age-groups and the treatment choices in the change of eGFR during the total 36-month period or the late phase with the IPTW-adjusted analysis.

The blockade of renal impairment has been considered as a crucial treatment benefit expected by ADX in patients with uPA. This notion was supported by the work by Hundemer et al. who reported that ADX in patients with PA might mitigate the risk for developing CKD, whereas treatment with MRA was associated with a higher risk for developing CKD when compared to essential hypertension^7^. We found that the eGFR during the late phase decreased to a greater extent in older patients that received the ADX treatment than the MRA-treated patients; however, this difference was not observed in younger patients. It should be noted that in both age groups, biochemical parameters of PA were higher in patients with ADX than patients with MRA. These differences probably reflect the real-world clinical practice in that disease severity can be a determinant for surgical treatment in uPA. Nevertheless, the IPTW-adjusted analysis raised the possibility that the ADX treatment could prominently decrease renal function compared to MRA-treated patients, even with patients that had equivalent clinical backgrounds. We also found a significant interaction between the age-groups and PA-specific treatments in the change of the eGFR with IPTW-adjusted analysis, which suggest that the impact of the treatment of choice in uPA on a patient’s renal function differs depending on the patient’s age.

It is widely known that glomerular hyperfiltration is followed by the development of proteinuria and renal damage due to renal sclerosis^26,27^. Previous studies have indicated that the initial decline in eGFR following PA-specific treatments is primarily caused by cancellation of hyperfiltration due to excessive aldosterone release^4–7^. We found that the initial decline in eGFR was smaller with the MRA treatment than with ADX, which could theoretically provide complete resolution of aldosterone excess. Nevertheless, normalization of glomerular hyperfiltration by ADX did not appear to prevent the development of renal injury during the late phase in older patients, compared to that in patients with MRA, or in younger patients with ADX (Fig. 2, Table 2). A recent JPAS study reported higher age was identified as an independent factor associated with a large initial decline in eGFR in patients treated with either ADX or MRA^18^. The findings suggest that older patients with PA are susceptible to developing glomerular injury from an early phase after PA-specific treatments. Aging is known to induce significant changes in the structure and function of the kidney^28–31^. The structural changes in kidneys could make older people prone to glomerular damage in response to a drastic change in renal hemodynamics. Therefore, rapid reduction in renal blood flow after PA treatment might have caused a decline in glomerular capillary pressure as well as irreversible glomerular damage in older patients. This supposition is supported by several clinical studies showing that older patients have a higher risk of irreversible glomerular damage due to an acute decline in glomerular filtration^32,33^. Together with the potency from ADX treatment that drastically reduces renal blood flow, it is conceivable that the negative influence of age on renal outcome may be greater with ADX treatment than with MRA treatment.

The current findings also suggest that the efficacy of ADX on BP regulation beyond MRA was obvious in younger patients with uPA but not in older patients. Although the temporal change in BP was not statistically different between ADX and MRA in both age groups (Fig. 3), the DDD except for MRAs after the specific treatments was lower with ADX than with MRA treatment in younger patients (Table 1). Notably, the treatment-associated difference was opposite in older patients (Table 1). Again, it should be noted that the biochemical severity is different between patients with ADX and with MRA, potentially interfering the pure comparison between the treatments. Nevertheless, the current findings provide an obvious contrast in the treatment effects of ADX between older and younger patients in BP regulation. Finally, it should be noted that the age-specific difference in the impact of the treatment on renal outcome is independent of that on the BP regulation, as BP control itself is not different between the treatments both in younger and older patients (Fig. 3).

There are several limitations in the present study. First, there are the treatment–associated differences in biochemical severity of PA that may interfere with the direct comparison between treatments. While our findings were strengthened by the consistency after the IPTW-adjusted analysis, future studies are required to validate that the ADX treatment is not beneficial in preserving renal function in older patients with uPA. Second, this was a multi-center retrospective observational study. As a strategy for specific treatments for PA was not pre-designed, the choice of treatments depended on the physicians. Particularly, Ohno Y et al. reported that the choice of ADX for uPA classified based on AVS was less frequent in Japanese than in European centers (78.2% vs 91.4%) from the international multicenter retrospective study on the Adrenal Venous Sampling Stats in Primary Aldosteronism (AVSTAT study)^34^. The cause of non-surgical treatment for the patients with uPA was more likely to be physician-derived, including good BP control, normokalemia, and the absence of adrenal lesions on imaging before AVS, in Japan^34^. Although the exact reason for this discrepancy remains unknown, it is conceivable that relatively mild uPA is more often diagnosed in Japan, compared with Europe. This country-specific difference may potentially influence on the generalizability of the present findings. Third, the medication adherence of MRA might be the potential confounding effect compared with ADX. Forth, we did not investigate the onset of CVDs, which are important complications for the vital outcomes of PA patients. Fifth, the 36-month follow-up period in this study is a relatively short period for assessing the long-term clinical outcomes. Together, prospective studies with long-term observation of outcomes including CVDs, will be required to determine the adequate specific treatments for PA in older patients. Finally, similar to most previous reports, we used only office BP measurement. Thus, the white coat effect might have influenced the BP data.

In conclusion, in younger patients with uPA, ADX could provide benefit by protecting chronic decline of renal function and resolving hypertension. In contrast, older patients with uPA experienced prominent post-treatment reduction of eGFR as well as poor improvement of hypertension. These findings suggest that older patients need careful monitoring after ADX. The limitations of this retrospective study hinder a direct comparison of the net treatment benefits; thus, future studies are needed to clarify if ADX can benefit older patients with uPA.
